# 729 A Post-Pandemic Educational Program for New Burn Nurses

**DOI:** 10.1093/jbcr/irad045.204

**Published:** 2023-05-15

**Authors:** Angela Klinkhamer, Megan Zynkian, Jeanne Lee

**Affiliations:** UCSD Medical Center, San Diego, California; UCSD Medical Center, San Diego, California; UC San Diego Health, San Diego, California; UCSD Medical Center, San Diego, California; UCSD Medical Center, San Diego, California; UC San Diego Health, San Diego, California; UCSD Medical Center, San Diego, California; UCSD Medical Center, San Diego, California; UC San Diego Health, San Diego, California

## Abstract

**Introduction:**

Transitioning into a nursing role within the burn center is clinically challenging and stressful. Due to the highly specialized nature of burn care, it is often difficult to find experienced nurses. Because of the shortage of experienced nurses, the burn center has been hiring many new graduate nurses. The COVID-19 pandemic greatly affected nursing schools, as clinical hours and experiences in the hospital were greatly reduced during peak outbreaks. Often, lab simulation and role playing were supplemented for clinical hours. In turn, nurses graduated with limited patient-nurse interactions and bedside experiences. From January 2020 through August 2022 our organization had an attrition rate of 27% for new graduate ICU nurses while the burn center saw a 25% attrition rate for new nurses. Our hypothesis is that an additional education program for new burn nurses will improve self-efficacy, knowledge and provide the support needed to promote long-term retention.

**Methods:**

An educational program was created and implemented for two nursing cohorts. Twelve additional hours of hands-on skills and discussions were added over the course of the standard twelve-week orientation period. A pre and post exam consisting of identical questions was given to all participating staff to assess baseline knowledge and effectiveness of education. Discussions within each cohort identified learning needs and areas where additional support could be offered. The General Self-Efficacy Scale was used to assess pre and post program implementation. Results were analyzed using an unpaired t test.

**Results:**

The average score in the eighteen-question pre-knowledge assessment exam was 72% and increased to 91% in the post-knowledge exam. The self-efficacy score increased from 1.68 to 3.36 (P value 0.0002). Based on self-efficacy results, nurses reported feeling more competent and less anxious about their new roles.

**Conclusions:**

The post-pandemic population of new graduate nurses face a grave disadvantage when starting a new role due to limited clinical experiences, lack of available rotations and a reduction in clinical hours. This may set them up for failure due to decreased self-efficacy. Providing additional support and a proactive approach to educating new nurses has proven to be a valuable experience in the burn center.

**Applicability of Research to Practice:**

Development of an educational program for new nurses was shown to improve self-efficacy and knowledge. Continuing this supplemental educational program for new nurses is beneficial to both new graduate and new burn nurses and has the potential to improve nursing retention.

